# The Use of Poly-d,l-lactic Acid (PDLLA) Devices for Bone Augmentation Techniques: A Systematic Review

**DOI:** 10.3390/molecules22122214

**Published:** 2017-12-13

**Authors:** Marco Annunziata, Livia Nastri, Gennaro Cecoro, Luigi Guida

**Affiliations:** Multidisciplinary Department of Medical-Surgical and Dental Specialties, University of Campania “Luigi Vanvitelli”, Via Luigi De Crecchio, 6, 80138 Naples, Italy; livia.nastri@unicampania.it (L.N.); gennarocecoro@gmail.com (G.C.); luigi.guida@unicampania.it (L.G.)

**Keywords:** poly-d,l-lactic acid, bone augmentation, systematic review

## Abstract

Poly-d,l-lactic acid (PDLLA) has been proposed in dentistry for regenerative procedures in the form of membranes, screws, and pins. The aim of this review was to evaluate the efficacy of bone augmentation techniques using PDLLA devices. A literature search was carried out by two independent and calibrated reviewers. All interventional and observational studies assessing the efficacy of bone augmentation techniques using PDLLA devices were included. Six studies were included. The relevant variability of design and methods impeded any qualitative or quantitative comparison. Ease of handling, absence of a re-entry phase, moldability of foils, and good soft-tissue response were appreciated characteristics of PDLLA devices. Some drawbacks such as the risk of membrane exposition, a prolonged adsorbability, and a tendency to a fibrous encapsulation of the PDLLA devices have been described, although the clinical significance of these findings is unclear. Clinical data about PDLLA devices for bone regeneration are very scarce and heterogenous. Well-designed randomized controlled trials comparing the use of PDLLA foils and pins with conventional membranes for bone regeneration are strongly encouraged in order to understand the real clinical benefits/drawbacks of this technique.

## 1. Introduction

Dental rehabilitation of partially or totally edentulous patients with oral implants has become a routine treatment modality in recent decades, with reliable long-term results [1,2,3,4,5], radically changing the field of dentistry and improving patients’ quality of life. However, unfavorable local atrophic conditions of the alveolar ridge, due to agenesis, periodontal disease, neoplasia, or traumatic events, may impair implant placement, and potentially compromise the long-term survival of implant rehabilitations from a functional and esthetic viewpoint.

In order to correct the unfavorable anatomy of atrophic sites, a number of alveolar ridge augmentation techniques have been developed, including autologous bone blocks from intra- and extra-oral donor sites, ridge splitting and expansion, distraction osteogenesis, sandwich osteoplasty, and guided bone regeneration (GBR) [6,7,8].

The use of membranes to promote bone regeneration following the principles of the guided bone regeneration (GBR) has become a standard of care in dentistry to correct bone defects at sites scheduled for implant placement, and the efficacy of such a procedure has been largely confirmed by long-term clinical results [9,10]. First membranes used for GBR application were made of expanded polytetrafluoroethylene (ePTFE), reinforced or not by titanium inserts (to increase their plasticity and dimensional stability), and provided excellent treatment results [11,12]. The ePTFE membranes, however, need to be removed in a second surgical stage. Furthermore, their efficacy is highly correlated to the wound healing course, being heavily jeopardized in case of wound dehiscence with consequent membrane exposure and infection [13,14]. To overcome such drawbacks, resorbable membranes made of animal-derived collagen or synthetic aliphatic polyesters were introduced, [15,16,17] with evident advantages in terms of simplified technique, no need of a second surgical stage and decreased patient morbidity. Nevertheless, in some cases, such membranes showed to be less effective than the non-resorbable ones, due to their reduced mechanical properties and a decreased barrier function, as a result of the rapid enzymatic degradation by macrophages and polymorphonuclear leukocytes [18].

Polylactic acid (PLA) is a semi-crystalline polymer with molecular weights of 180,000 to 530,000, a melting point of about 174 °C, and glass transition temperature of 57 °C. Depending on the l and d configuration, it can exist in several distinct forms, such as poly-l-lactide (PLLA) and poly-d-lactide (PDLA), and it is also degraded via hydrolysis [19]. By adding d-isomers into an l-isomer based polymerization system of PLA, polymer chains widen and cannot be packed as tightly as PLLA polymer chains. The resulting material, the poly-d,l-lactic acid (PDLLA), is characterized by biomechanical, thermal, rheological, and biological properties which may be modulated on the basis of the different proportions of l- and d-isomers composing the different PDLLA formulations. d-isomer, indeed, is characterized by a more rapid resorption and a less crystalline structure, whereas the l-isomer is characterized by a higher crystallinity and a less rapid resorption. Regarding the rheological properties of the material, the shear viscosity of the polymer increases with increasing l-isomer in the l/d-isomer mixture because of the increasing crystallinity of PLA [20]. Also, the glass transition temperature increase with increasing the amount of the l-isomer [21]. This material, like other resorbable materials, degrades and resorbs in two phases [22]. During the first phase, water molecules hydrolyze the long polymer chains into shorter fragments. The second phase consists of a physiologic response of the body in which macrophages phagocyte and metabolize the short fragments which subsequently enter the citric acid cycle [23,24,25], and are transformed into carbon dioxide and water, subsequently excreted from the body, mainly through respiration.

PDLLA has been proposed in dentistry for regenerative procedures in form of fluid membranes and solid foils, screws, and pins. PDLLA fluid membranes (Atrisorb, Atrix Labs, Fort Collins, CO, USA) mainly used for guided tissue regeneration (GTR), in some case for GBR, transforms to a solid barrier when contacted with water or aqueous solutions and solidifies in situ. This fluid membrane is composed of 37% of a liquid polymer of lactic acid that is dissolved in 63% *N*-methyl-2-pyrrolidone; polymer drops are mixed with saline drops in a ratio of one drop of saline every two drops of polymer; then a rapid mixing of polymer and saline is performed in order to drive off the *N*-methyl-2-pyrrolidone carrier. The solid membrane, pins, and screws (Resorb-X, KLS Martin, Tuttlingen, Germany), mostly used for GBR, are composed of a 50/50 mixture of d and l-isomers of PLA and is characterized by an extended resorption time and increased mechanical properties [26,27]. A number of preclinical studies have investigated the regenerative features [28], the biological properties [29,30,31] as well as the physical–mechanical properties [32,33,34] of these PDLLA devices. On the contrary, very few studies have evaluated the clinical performance of PDLLA devices for bone regeneration, so that a comprehensive understanding of their potential role in this field is still lacking.

The aim of this review was to evaluate the efficacy of bone augmentation techniques using PDLLA devices, through a systematic search of the current available scientific literature.

## 2. Materials and Methods

The search strategy used in this systematic review was based on the PRISMA (Preferred Reporting Items for Systematic reviews and Meta-Analyses) guidelines (http://www.prisma-statement.org) [35]. Clinical questions were formulated according to the PICO framework for evidence based practice [36]. The focused question was: “What is the efficacy of bone augmentation techniques using PDLLA devices compared to traditional materials in terms of qualitative and/or quantitative changes of hard tissue at the augmented sites?”

A literature search was carried out on November 2017 by two independent and calibrated reviewers (M.A. and G.C.) using an ad hoc created search string in the database of the National Library of Medicine MEDLINE/PubMed, in the Cochrane Central Register of Controlled Trials, and in the ClinicalTrials.gov website. No language restriction was applied. All interventional studies (either randomized or non-randomized controlled clinical trials) and observational studies (either analytical or descriptive) assessing the efficacy PDLLA devices to bone augmentation techniques were considered. Sinus lift and alveolar preservation procedures were also included. Preclinical studies, technical reports, case reports, review, and conference abstracts were not considered. No restriction on age or number of patients, as well as on the follow-up duration was considered. Studies regarding osteosynthesis techniques and periodontal regeneration were not included. All full text studies were carefully read and analyzed for the eligibility criteria (inclusion/exclusion). Reported results and data were analyzed for qualitative and/or quantitative changes of hard tissue at the augmented sites. The main features of the bibliographic search are reported in Table 1. 

## 3. Results

**Figure 1:**
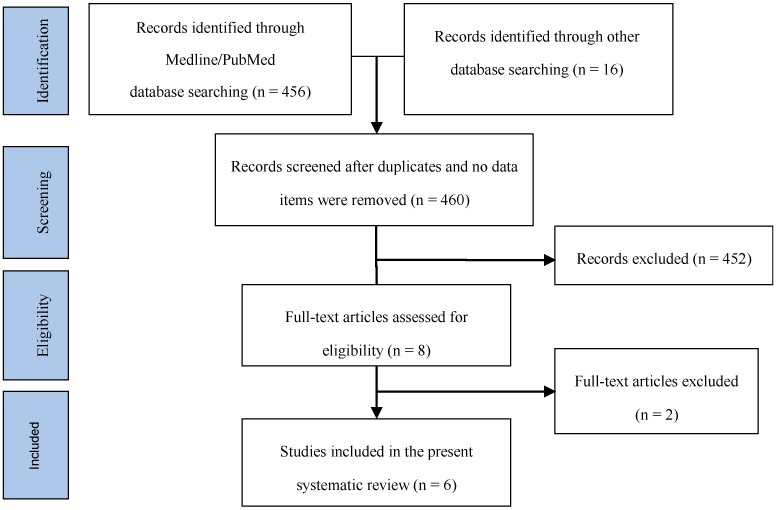
Flow diagram (PRISMA format) of the screening and selection process.

456 items in MEDLINE/PubMed and 16 in other sources were found after the initial search (14 items in the Cochrane Library, 2 in the ClinicalTrials.gov database). After duplicates were removed, 460 records remained. They were screened on the basis of titles and abstracts for inclusion/exclusion criteria, so that 452 studies were excluded. After full text reading of the remaining eight papers, two of them were excluded because they used a polylactic material different from PDLLA. At the end of the process, six studies published between 2001 and 2016 were included in this systematic review (Figure 1).

The main characteristics of the included articles are reported in Table 2.

In the study of Rosen & Reynolds (2001) titled “Guided bone regeneration for dehiscence and fenestration defects on implants using an absorbable polymer barrier” [37], PDLLA fluid membranes (Atrisorb) were used to treat peri-implant defects at the time of implant insertion. The purpose of the report was to present consecutive clinical experiences with a poly(d,l-lactide) polymer barrier in combination with a composite bone replacement graft for GBR of dehiscence/fenestration defects during implant placement. Two implant sites requiring ostectomy for exposure of the fixture permitted histologic evaluation of the new bone formation. Nine patients, for a total of 11 peri-implant defects, were treated with a composite graft of demineralized freeze-dried bone allograft (DFDBA) mixed with freeze-dried bone allograft (FDBA) in a 1:1 ratio. The graft was then covered with the PDLLA membrane that overlapped the adjacent osseous structures for 2 to 3 mm. Photographs were taken of the implant and GBR procedure, and clinical measurements were made of the dehiscence/fenestration defects treated. The osseous defects treated included eight dehiscence and three fenestration defects. The success or failure of the GBR procedure was assessed at the second-stage surgery when flaps were reflected to place healing abutments. Complete success was defined as coverage of all threads or exposed implant surface. Partial success was deemed as incomplete coverage of most threads or exposed implant surfaces with a maximum of two threads or 2 mm of implant surface left uncovered. Failure was defined as no coverage beyond two threads or 2 mm of implant surface. The length of the defects ranged from 2 to 13 exposed threads with an average of 8.5 exposed threads. With the exception of one dehiscence lesion that had partial coverage, all other defects had complete coverage of the implants, representing a 90.9% success rate. The barriers were well tolerated by the tissue with no sites demonstrating any type of tissue reactions or infection. The histologic evaluation of biopsy specimens revealed the presence of viable bone and residual graft particles. Notably absent in all specimens was evidence of an inflammatory cell infiltrate. The authors emphasize some advantages of this fluid barrier. The polymer system permits repair or modification of the barrier if it is inaccurately cut or trimmed, since additional material can be added to the existing barrier. The barrier’s final consistency is hard and rigid, adding to its space maintenance capabilities. It takes approximately 5 to 6 months for significant degradation of the barrier to begin, and it is probably completely absorbed by 12 months. The authors also highlight some limitations of this material. The final consistency of the barrier is hard and firm, making it susceptible to fracture and premature loss in the presence of untoward occlusal forces. This event occurred in a patient at one implant site, although the clinical outcome was still favorable. In the presence of large defects, premature loss of the barrier might substantially compromise the regenerative outcome. Furthermore, the exposure of the poly(d,l-lactide) barrier may also lead to a premature loss, with regenerative consequences related to the maturational stage of the wound healing process. 

In the study of Raghoebar et al. (2006) titled “Resorbable Screws for Fixation of Autologous Bone Grafts” [38] PDLLA pins for fixating autologous bone grafts were used. Eight patients were included in this split mouth RCT. In all cases, a two-stage procedure (first stage—bone grafting; second stage—placement of implants) was performed bilaterally. Randomly, the grafts were fixed to the alveolar bone with 1.5 mm large titanium screws at one side (Martin Medizin Technik) (control) and with 2.1 mm large PDLLA resorbable screws (Resorb X, Martin Medizin Technik) on the other side (test). The bone blocks were fixed with two titanium screws on one side or two resorbable screws on the other side, in a region where no implants were planned to be inserted. The bone width was measured with a caliper. The bone grafts were covered also with a resorbable membrane. After a healing period of at least three months, the implant placement procedure was performed. After reflecting the mucoperiosteal flap, the width of the reconstructed alveolar crest was measured and the titanium screws were removed. Using the template for insertion of the implants, in order to avoid areas in which the implants will be inserted, a bone biopsy was taken with a trephine bur including one resorbable screw. In all cases, the bone volume was sufficient. Six months after insertion, the implants were uncovered and another bone biopsy was taken from the area with the other resorbable screw included. The width of the alveolar ridge was between 2 and 4 mm before augmentation and 6 and 8 mm after reconstruction. There was no difference in the bone width between the two sides. Neither at three nor at nine months were observed differences between both sides. A total of 56 implants were placed in the augmented maxillae. No implants were lost during the follow-up (22.2 ± 4.3 months). Microscopically, three and nine months after reconstruction of the maxilla with autologous bone (most of) the PDLLA material was still visible. The material was shown to be biocompatible as no inflammatory response was observed both at the three- and nine-month evaluation periods. It must be emphasized that PDLLA screws did not interfere in the incorporation of the grafts and on bone viability and/or quality. The PDLLA screws, whose contours were clearly visible in all biopsies, were encapsulated by a thin and immature fibrous tissue capsule containing many giant cells in direct contact with the PDLLA material as well as infiltrating in areas with fragmented PDLLA. PDLLA particles were observed within the giant cells. The adaptation of the biodegradable screws for fixation of the bone grafts resulted as uncomplicated as with titanium screws. The authors emphasize the advantages of using a biodegradable material for osteosynthesis over metals: no need of material removal; no risk that the head of the screw can protrude as the bone grafts heal and bone resorption occurs, causing discomfort, soft-tissue dehiscence, infection, and even graft failure [39]; no risk of compromission of the implant area, possible when the metallic screws, covered by bone, were positioned in the region where the implants had to be inserted. Furthermore, although the small sample size, the authors highlight that no significant difference, and even no tendency, in remodeling of the grafts fixed with either titanium or resorbable screws could be observed. However, they also underline a significant finding concerning PDLLA resorbability. After nine months, in fact, screw remnants were still present, surrounded by foreign body cells. Although no significant inflammatory signs were visible and no patient presented any kind of clinical or radiographic manifestation suggestive of a more severe inflammatory reaction in response to PDLLA than titanium screws, this finding suggests caution using this resorbable material as fixation devices when endosseous implant insertion is planned. The presence of the connective tissue in bone–screw interface does not represent an obstacle to the placement of implants. However, whether the maintenance of the polymer in this specific experimental period can interfere during the preparation of the implant site and the effects of this manipulation cannot be predicted. Considering this observation, the authors affirm that the resorbable screws would be of great use, especially in large reconstructions, if they can be positioned in areas where they certainly will not interfere with the planning of the endosseous implants.

In the study of Santana & de Mattos (2009) titled “Efficacy of Vascularized Periosteal Membranes in Providing Soft Tissue Closure at Grafted Human Maxillary Extraction Sites” [40], a fluid PDLLA membrane (Atrisorb) a total of 56 post-extractive sites in 22 patients with a split mouth design was used. The objective of this study was to evaluate the efficacy of a flap design, based on the extension of palatal tissues, to obtain and maintain soft tissue coverage over grafted extraction sockets with or without the use of an absorbable fluid PDLLA membrane. In the control side, post-extractive sites were treated with a 1:1 mixture of decalcified freeze-dried bone allograft material (DFDBA) and an inorganic bovine bone mineral (Bio-Oss™), covered with the vascularized periosteal membrane. The test side received the same graft material and the vascularized periosteal membrane, but the sites were also covered with a fluid PDLLA membrane. The measurements of the crestal bone width were made from the crest of the buccal bony wall to the crest of the palatal bony wall, with a calibrated periodontal probe at a point corresponding to half the mesiodistal socket diameter. The measurements were made at two times: at the time of the surgery, just prior to the graft placement, and six months after tooth extraction, after the elevation of full-thickness buccal and palatal flaps to expose the alveolar crest. Baseline measurements of the ridge dimensions (BCW) revealed a mean of 7.9 ± 0.9 mm for the control sites and 8.2 ± 0.8 mm for the experimental sites. Repeated measurements obtained six months after tooth extraction showed that the test and control groups performed equally well. Mean BCW in the controls was 6.9 ± 0.7 mm, and in the experimental sites it was 7.5 ± 0.8 mm. This small difference did not reach statistical significance. The results of the present study showed that both techniques were effective in obtaining and maintaining soft tissue closure over grafted extraction sockets. The presence of a barrier membrane did not increase the incidence of soft tissue dehiscence, and elevated levels of soft tissue closure were maintained throughout the study period. Membrane exposure was observed in 7% of the test sites. In view of the reported variations in frequencies of barrier exposure following GBR procedures reported in the literature, as well as findings of reduced bone regeneration in cases of early membrane exposure [13,41] the results of the present study suggest that the vascularized periosteal membrane technique holds promise in optimizing the clinical results of GBR protocols. No signs of erythema, edema, swelling, or suppuration were noticed in the soft tissues of the experimental group. Soft tissue healing at sites treated with the absorbable membrane did not appear to be different from that observed at the control sites. Moreover, in the few areas where the membranes were exposed during healing, the exposed parts of the membrane margins disappeared concurrently with healing of the soft tissues. No signs of infection or suppuration were observed in exposed membrane sites, suggesting that the material was well tolerated by the tissues. The authors conclude that this material, in addition to GTR, may also be suitable for alveolar ridge preservation in conjunction with composite bone grafting and that, within the limitations of the study, the vascularized periosteal membrane was an adequate choice for achieving and maintaining complete soft tissue coverage and healing by primary intention of grafted extraction sockets in humans. The procedures allowed for optimal levels of complete coverage of absorbable membranes during healing; however, the use of an absorbable membrane provided no additional clinical benefits.

In the study of Burger B. W. (2010) titled “Use of Ultrasound-Activated Resorbable Poly-d-l-lactide Pins (SonicPins) and Foil Panels (Resorb-X) for Horizontal Bone Augmentation of the Maxillary and Mandibular Alveolar Ridges” [42] have been used. The aim of this study was to determine the efficacy of 50% poly-d-lactide and poly-l-lactide pins (SonicPins, KLS Martin) and foil panels (Resorb-X, KLS Martin) in conjunction with a particulate bone allograft (Puros, RTI; Biologics; Alachura, FL, USA) in patients in need of at least 3 mm of horizonal bone augmentation in multiple sites of maxilla and mandible. Although the authors also report in the text the use of both a PDLLA membrane and a collagen membrane (OsseoGuard GTM, Biomed, 3i West Palm, FL, USA), in conjunction with the bone graft for the resolution of horizontal ridge defects, the paper is not clearly structured with a controlled study design (experimental groups are not punctually described and comparative outcome measures are not reported at all). The authors extensively describe the surgical procedure, together with the advantages of the PDLLA pins and membranes. The sites for pin placement were prepared with a drill that penetrated at least through the outer bony cortex; the pin is pushed into a funnel-shaped pilot hole with sonic frequency vibration: the edges of the pin rub on the bone, causing friction to bring the polymer to a liquid state. The pilot hole is smaller than the pin, causing the polymer to flow into the trabecular spaces of the bone. When the sonic frequency is stopped, these liquefied portions become hard again in seconds. When welding the resorbable SonicPins to the bone, the pins were extremely easy to handle and it took very little time to place them, even in the posterior maxilla, where access is limited. The sonic pins were very secure, did not fracture or break, and did not have to be replaced with a larger diameter pin. It was also easy to shorten the pins by resonication. The Resorb-X foil panels did not need any additional support: although it was extremely thin, the foil provided its own rigidity and support after cooling. Once the foil is heated, it can be adapted to the bone surface and shaped to the desired position. Once cooled, the material turns rigid again and reliably retains its shape. The foils used in this study were 0.1 mm thick, which provided excellent support for the particulate bone graft and to tent the mucoperiosteum. Tissue response to the PDLLA devices was unremarkable: there was no evidence of prolonged inflammation, swelling, or discomfort at any of the operative sites. When comparing preoperative with postoperative computer tomographic scans, there was an approximately 3 mm increase in bone width. Upon entering the grafted sites, there was sufficient bone width to place the implants. In conclusion, the authors underline some advantages with the use of these resorbable PDLLA foil panels and pins: the easy handling and placing of pins and foil, the excellent support provided by pins and foils to the mucoperiosteum, above the underlying particulate bone grafting; the possibility of shaping foils to the desired dimension for adequate augmentation.

We choose to consider, in a more extensive way, also the maxillary sinus elevation among the bone augmentation procedures. For this reason, we included in the present review two recent studies on the use of such devices for the maxillary sinus elevation. 

In their study titled “Elevation of the Maxillary Sinus Membrane for De Novo Bone Formation: First Results of a Prospective Study in Humans” [43], Lie et al. (2015) used PDLLA membranes to perform an elevation of the maxillary sinus membrane. The aim of this study was the clinical and histological investigation of bone regeneration and integration of a well characterized inorganic bovine bone substitute material (Bio-Oss™; Geistlich Biomaterials GmbH, Wolhusen, Switzerland) mixed with autogenous bone in comparison to elevation of the sinus membrane alone. Five patients were included in this split mouth RCT. One maxillary side was augmented using the standard augmentation procedure with a mixture of autogenous and xenogenous bone. On the contralateral side the Schneiderian membrane of the maxillary sinus was lifted and stabilized with a degradable perforated PDLLA membrane (Resorb-X, KLS Martin) and PDLLA pins (SonicPins, KLS Martin). Six months after the sinus floor augmentation, six implants in each patient were positioned, with a total of 30 implants. Pre- and post-operative cone-beam computed tomography (CBCT) scans where compared and the gain in bone volume was measured using a digital program. During the implant insertion, a trephine bur was used for the preparation, so it was possible also to take bone probes for histological evaluation. After six months, sufficient bone was present in both sides at the radiological evaluation. However, the non-augmented side showed less opacity, which suggested less bone density or less presence of mature bone. However, primary stability of the implants was achieved in all cases independently of the augmentation technique applied. All bone specimens showed new bone formation. However, the bone of the experimental side was less organized and immature. The bone biopsies of the conventional side showed as expected the combination of xenogenous bone, embedded in bone marrow and newly formed bone. The biopsies from the experimental side showed new bone formation and osteoblast and osteoclast activity as signs of an active bone forming and remodelling process. Implant success rates were 100% on both sides one year after the prosthodontic rehabilitation. All implants could be loaded by prostheses in a conventional way (bar-retained overdentures) and are successful after the follow up time. All patients reported comfort with the dentures and were fully satisfied. Based on the randomization, patients did not mention differences between both sides of the maxilla. Prosthetic follow up differed from 12 months post-loading in the first study patient to 6 months post-loading in the last patient. The authors concluded that the use of a resorbable PDLLA membrane proved to be a good and reliable technique to create a stable elevation of the sinus membrane. The major advantage of the technique presented is the absence of any kind of donor site morbidity (autogenous bone) and there is no risk of infectious proteins (xenogenous bone substitutes). PDLLA membrane did not interfere with the bone forming process, which resembles a callus-based bone formation in a space surrounded by bony walls. The perforated mesh design of the membranes used seems to be logical to allow vascular ingrowth and the material degrades without generation of any crystalline remnants [44].

In the study of Gocmen et al. (2016) titled “Hyaluronic Acid versus Ultrasonic Resorbable Pin Fixation for Space Maintenance in Non-Grafted Sinus Lifting” [45], PDLLA pins were used to perform an elevation of the sinus membrane. The aim of this study was to compare hyaluronic acid with ultrasonic resorbable pins for their ability to maintain intrasinusal space. 10 patients were included in this split mouth RCT. One maxillary side was augmented with the elevation of the sinus membrane and the positioning of a hyaluronic acid matrix (Hyaloss Matrix, Anika Therapeutics, Bedford, MA, USA). On the contralateral side, the Schneiderian membrane of the maxillary sinus was lifted and stabilized with a degradable PDLLA pin (Sonic-Pin Rx, KLS Martin, Muhlheim, Germany) of 11 to 17 mm in length, fixed 2 mm above the bone window used to perform the sinus lift. CBCTs were taken before the surgery and six months after. The mean postoperative height of alveolar bone values were 9.6 ± 3.4 mm on the Sonic-Pin Rx side and 6.4 ± 2.6 mm on the hyaluronic acid side. The postoperative mean height of alveolar bone values were significantly greater than the preoperative mean values on the two sides. The mean increases in height of alveolar bone and reduction of sinus volume on the Sonic-Pin side were significantly greater than those on the hyaluronic acid side. Patients were treated with two implants in each side, for a total of 40 implants placed. The bone quality at the moment of the implant insertion was of type II or higher in all the sites. A late-loading protocol was followed, and the mean healing time for abutment loading was 16.4 ± 2.5 weeks. At six months, all implants were clinically stable, and the definitive prostheses were functional, with a survival rate of 100%. In conclusion, there was sufficient bone height to eventually place the implant in the right and left sides of all patients. The two techniques yielded predictable outcomes in implant survival and bone quality. However, the height of alveolar bone and reduction in sinus volume were considerably greater on the PDLLA pin sides. This might be due to the features of the resorbable pins as a rigid barrier or the weak mechanical properties of bone formation on the hyaluronic acid side.

## 4. Discussion and Conclusions

PDLLA devices have been proposed for bone augmentation procedures in different forms, based on the advantageous possibility of enhancing the biomechanical, rheological, thermal, and biological properties of this material by modulating the ratio of D and L isomers in its formulations. Despite a larger presence of pre-clinical studies as well as of clinical application of PDLLA devices for other applications, such as osteosynthesis techniques, clinical data on bone augmentation procedures are very scarce. Only six clinical studies about PDLLA devices for bone augmentation were found. One of them was a descriptive study (case series), five were controlled studies (three randomized). PDLLA was used in the form of PDLLA foils, screws, and pins. An evident heterogeneity in clinical indications and augmentation techniques used with the aid of the PDLLA devices was found: alveolar preservation of post-extraction sites by PDLLA foils, bone block fixation by PDLLA screws, sinus lift using PDLLA foils or pins, and correction of peri-implant dehiscences and fenestrations by PDLLA membranes and pins. Such a variability impeded any qualitative or quantitative comparison among the studies. About the clinical use of PDLLA devices the authors emphasize the ease of handling, absence of a re-entry phase, moldability of foils, good soft-tissue response. The authors point out also some drawbacks including the risk of membrane exposition, a prolonged adsorbability, and, from a histological point of view, a tendency to the fibrous encapsulation of the PDLLA devices (although the clinical significance of this finding is unclear).

In conclusion, the results from the available studies are inconclusive about the clinical efficacy of PDLLA devices for bone augmentation procedures, mainly because of the high heterogeneity and the lack of sufficiently powered and appropriately designed trials. Well-designed randomized controlled trials of sufficient size comparing the use of PDLLA foils and pins with conventional membranes for bone augmentation procedures are strongly encouraged in order to understand the real clinical benefits/drawbacks of this technique.

## Figures and Tables

**Table 1:** Systematic search strategy.

Systematic Search Strategy
**Search string:** (pdlla OR “poly-d,l-lactic acid” OR “poly(l,d-lactide)” OR “poly-d-l-lactide” OR “poly-d,l-lactide” OR “poly(l/d-lactide)” OR “poly(l,d-lactic acid)” OR “poly-d,l-lactide acid” OR “Atrisorb” OR “Resorb-X” OR “SonicWeld” OR “SonicPins”) AND (oral OR dental OR bone) AND (membrane OR shell OR pin OR barrier OR screw OR mesh) **Filter:** Humans **Language:** No restrictions **Electronic databases:** MEDLINE/PubMed, The Cochrane Central Register of Controlled Trials (CENTRAL), ClinicalTrials.gov **Inclusion criteria:** - Study design: interventional and observational studies - Population: partially edentulous patients - No restriction on age or number of patients - Healthy individuals (no systemic diseases, no medications affecting platelet and bone functions) - Intervention: bone augmentation using PDLLA devices - Comparison (if appliable): any other augmentation technique or material - Outcome: qualitative and/or quantitative changes of hard tissue at the augmented sites. - Follow up duration: any **Exclusion criteria:** - Studies regarding osteosynthesis techniques or periodontal regeneration - Conference abstracts, pre-clinical studies, technical reports, and reviews.

**Table 2:** Main characteristics of the included articles. RCT: randomized controlled trial; CT: controlled trial; ND: not definable; CBCT: cone beam computed tomography.

Autor, Year	Design	Patients (Sites)	Groups	Follow-Up	Results	Conclusions
Gocmen et al., 2016	RCT, Split mouth	10 (20)	Test side: elevation of the maxillary sinus membrane and placement of a PDLLA pin. Control side: elevation of the maxillary sinus membrane and placement of a hyaluronic acid matrix.	6 months	Significantly higher postoperative mean values of alveolar bone height on both sides compared to the preoperative ones. Significantly greater increase of alveolar bone height and reduction in sinus volume on the test side compared to the control side. 100% implant survival rate at both sides.	There was sufficient bone height to eventually place the implant in both sides of all patients. The two techniques yielded predictable outcomes in implant survival and bone quality. However, the height of alveolar bone and reduction in sinus volume were considerably greater on the PDLLA pin sides.
Lie et al., 2015	RCT, Split mouth.	5 (10)	Test side: sinus lift using exclusively a PDLLA membrane fixed with PDLLA pins. Control side: sinus lift using a mixture of inorganic bovine bone substitute material and autologous bone.	6 months	Bone formation revealed by CBCT at both sides, but less radiopacity shown at the test sides. Vital new bone at the histological analysis, although less organized and immature, at the test side and a mixture of autogenous and residual bone substitute at the control side. 100% implant survival rate at both sides.	The PDLLA membrane proved to be a good and reliable technique to create a stable elevation of the sinus membrane and seemed not interfere with the bone forming process.
Burger BW, 2010	CT, Parallel groups	ND	Test group: horizontal bone augmentation with PDLLA membrane and pins + particulate bone allograft. Control group: horizontal bone augmentation with collagen membrane + particulate bone allograft.	ND	About 3 mm increase in bone width measured by computed tomography. No clinical difference in the nature of the grafts and in the bone density between the test and control groups. Unremarkable tissue response. No evidence of prolonged inflammation, swelling, or discomfort.	There are many advantages to using the resorbable PDLLA pins and foil panels for augmenting alveolar ridge defects: the handling and placing the pins and foil is easy and provides excellent support for the underlining mucoperiosteum for particulate bone grafting.
Santana & de Mattos, 2009	CT, split mouth	22 (56)	Test side: post extractive sites treated with decalcified freeze-dried bone allograft and inorganic bovine bone mineral graft + vascularized periosteal membrane + fluid PDLLA membrane. Control side: post extractive sites treated with decalcified freeze-dried bone allograft and inorganic bovine bone mineral graft + vascularized periosteal membrane.	6 months	Ridge dimension change showed a small not statistically significant difference in favor of the PDLLA membrane. 7% membrane exposition rate at the test sites. No signs of erythema, edema, swelling, or suppuration in any test or control site. Comparable soft tissue healing at both test or control sites.	Fluid PDLLA membrane may be suitable for alveolar ridge preservation in conjunction with composite bone grafting. Within the limitations of the present study, it was concluded that the vascularized periosteal membrane was an adequate choice for achieving and maintaining complete soft tissue coverage and healing by primary intention of grafted extraction sockets in humans. The procedures allowed for optimal levels of complete coverage of absorbable membranes during healing; however, the use of a fluid PDLLA membrane provided no additional clinical benefits.
Raghoebar et al., 2006	RCT, split mouth	8 (16)	Control side: autologous bone graft fixed with titanium screws. Test side: autologous bone graft fixed with resorbable PDLLA screws.	3–9 months	Comparable increase (about 4 mm) of the alveolar ridge width in both test and control sides. PDLLA material still visible at the histological analysis, not in direct contact with the bone and surrounded by fibrous tissue, thin and immature, with a lot of giant cells; no necrosis, hemorrhage, or significant inflammatory response. 100% implant survival rate at both sides.	Resorbable screws can be used as fixation devices for autologous bone grafts. However, care must be taken when using these screws because of the presence of the polymer (remnants) after nine months, which could interfere in the sequence of the treatment with endosseous implants.
Rosen & Reynolds, 2001	Observational descriptive study (case series)	9 (11)	11 implants placed in combination with a composite graft of demineralized freeze-dried bone allograft (DFDBA) mixed with freeze-dried bone allograft (FDBA) in a 1:1 ratio, covered with a fluid PDLLA membrane.	4 to 8.5 months	Complete coverage in 10/11 sites (success rate 90.9%). No site had inflammatory reactions or infection. Vital bone, at the histologic evaluation, in close amalgamation with graft particles, without inflammatory infiltrate.	Fluid PDLLA membranes can be used in conjunction with a bone graft to treat peri-implant dehiscences and fenestrations.
